# Ethnomedicine in Himalaya: a case study from Dolpa, Humla, Jumla and Mustang districts of Nepal

**DOI:** 10.1186/1746-4269-2-27

**Published:** 2006-06-02

**Authors:** Ripu M Kunwar, Bal K Nepal, Hari B Kshhetri, Sanjeev K Rai, Rainer W Bussmann

**Affiliations:** 1 Center for Biological Conservation, Nepal; 2 University of Hawaii, USA

## Abstract

Traditional plant use in Nepal has been documented for millennia. The importance of plants as medicine has not diminished in any way in recent times, and traditional medicines are still the most important health care source for the vast majority of the population.

This paper examines the ethnobotany and traditional use of plants extracted from the vulnerable alpine zone in the Dolpa, Humla, Jumla and Mustang districts of Nepal.

The results of this ethnobotanical study indicate that a very large number of plant species is used as traditional medicines. There were 107, 59, 44 and 166 species of ethnomedicinal importance in surveyed areas of Dolpa, Humla, Jumla and Mustang district respectively. Of these, 84 common species, used at least in two districts, were selected to enumerate their ethnomedicinal properties. The 84 species belonged to 75 genera and 39 families.

The commonest species in this pharmacopoeia were: *Allium wallichii, Cordyceps sinensis, Dactylorhiza hatagirea*, and *Rheum australe*. A total of 21 species were most common in three districts and 59 in two districts. The genera *Aconitum, Allium, Arisaema, Berberis, Corydalis, Gentiana, Hippophae, Juniperus *and *Rhododendron *each possessed two species with ethnomedicinal use. Labiatae was the most medicinally important family with five species used, followed by Araceae, Compositae, Liliaceae, Polygonaceae, Ranunculaceae, Scrophulariaceae and Umbelliferae, each contributing four species.

## Background

The use of plants and plant products as medicine can be traced as far back as the beginning of human civilization. The earliest record of medicinal plant use in the Himalayas is found in the *Rigveda*. This work was written between 4500 BC and 1600 BC, is supposed to be the oldest repository of human knowledge and describes 67 plants [1]. After the *Rigveda*, *Ayurveda *(the foundation of science of life and the art of healing of Hindu culture) describes the medicinal importance of 1200 plants. The *Charak *or *Caraka Samhita *(900 BC) and *Susruta Samhita *(500 BC) enumerate the art of surgery, therapeutics and medicines in detail on the basis of *Atharvaveda *[2]. The knowledge of using these systems was accessed by Nepali *Vaidhyas *and *Kabirajs *as early as about 879 AD [3]. Therefore, the Ayurvedic physicians were incorporating medicinal plants in traditional Ayurvedic formulations from early on and the Ayurvedic system is reputed all over the Indian sub-continent since time immemorial [4,5]. In addition to the Ayurvedic system, a large number of plants are also used in different other traditional health systems, including Homeopathic, Amchi (practice of Tibetan traditional medicine), Chinese [6,7], and folklore [8]. Plants for health care were also processed, exchanged and form part of secular trade systems [9-11]. The use of locally available medicinal plants is often an economically inevitable alternative to expensive western medicines [12].

A hand written herbal encyclopedia *Bir Nighantu *or *Bir pharmacopoecia *was compiled by Pandit Ghana Nath Devkota under the instruction of Bir Samser, the former prime minister of Nepal (1885–1901 AD). An elaborated account is found in *Nepali Nighantu *written by Kosh Nath Devkota published by the Royal Nepal Academy in 1969 [5]. This encyclopedia covers 750 plants in detail and is probably the first written effort towards a compilation of the traditional knowledge about medicinal plants of the country. The earliest published work dealing with the medicinal plants of Nepal was published in 1955 [13].

Medicinal plants from Nepal were traded across the borders to Tibet as early as 600 AD [14]. Presently, over 90% of the total export from Nepal is to India and mostly in crude form [15]. Conservative estimates of the annual Nepalese alpine and sub-alpine medicinal plant vary from 480 to 2500 tons, with a total harvest value of US$ 0.8–3.3 million [16]. Presently the value is much higher already (Table 1).

**Table 1:** Amount of important medicinal plants exported from Nepal (in tons)

**Species**	**1999**	**2000**	**2001**	**2002**	**2003**	**2004**	**Average**
*Acorus calamus*	20	12	11	9	6	10	11
*Cordyceps sinensis*				.0031	.0045	.0756	.0277
*Delphinium himalayai*	.5	2.1	6.4	12	2	2	4.3
*Morchella conica*				.93	5.1	1.1	2.4
*Nardostachys grandiflora*	29	83	46	254	45	208	111
*Neopicrorhiza scrophulariiflora*	35	46	39	.2	.3	5.2	21
*Valeriana jatamansii*	33	34	20	28	42	88	41
*Zanthoxylum armatum*	355	361	533	1006	650	365	545

Source: *Department of forest, Ministry of forest and soil conservation, Kathmandu, Nepal*

Medicinal and aromatic plants are local heritage of global importance [17]. Total 60% of the population of world and 80% of the population in developing countries rely on traditional medicine, mostly plant drugs, for their primary health care needs [18]. An account of 70% of the population of India [19], 80% of Pakistan [20] and 80% of Nepal are dependent on traditional plant based medicines. Medicinal and aromatic plants help in alleviating human suffering and are widely used as additives, beverages, cosmetics, sweeteners, bitters, spices, dying agents and insecticides. They are found throughout Nepal, from the plains to the high Himalayas, with the greatest concentration in the tropical and arid zones. Nepal recognizes about 1624 plant species as having medicinal and aromatic values, Sri Lanka about 1400, India about 2500 and China about 5000 [17,21,22]. A tentative list of the alpine flora of the Nepal Himalaya consists of 1227 species in 317 genera [23] including 114 and 45 medicinal plants respectively from the subalpine and alpine zones [1]. The Himalayan region shows the highest richness for endemic species and medicinal herbs [24].

Due to changing life, perception and lifestyle changes of the forest dwellers, as well as commercialization and socio-economic transformation on a global scale, there is a general observation that the plants are exacerbated and that indigenous knowledge on resource use is being degraded severely [25,26]. Due to lack of organized, sustainable cultivation based on scientific data and lacking awareness of social factors influencing plant use and market, no proper management of traditional medicines is in place, and the numbers of these plants are decreasing at an alarming rate [27]. Medicinal herbs are regarded as free commodity (zero private cost) to be collected from nature [28]. Ethnomedicinal studies are a suitable source of information regarding useful medicinal plants that can be targeted for domestication and management [29]. In this context the human interferences is considered an essential ecological factor for managing the plant resources and ethnobotanical knowledge. The present study therefore aims at highlighting the ethnomedicinal uses of plant resources of the Himalaya regions.

## Study area

The study area comprises Chandannath, Depalgaun, Garjyang and Khalanga villages of Jumla district; Chhuksang, Samar and Lomanthang villages of Mustang district; Mimi, Melcham and Dharma villages of Humla district and Dunai, Juphal, Raha, Tripurakot and Phoksundo villages of Dolpa district. Some villages of Dolpa and Mustang districts respectively represent the protected areas: Shey-Phoksundo National Park and Annapurna Conservation Area. All the areas are above 2200 masl and extend up to 6800 masl.

The major ethnic groups of the study area are Gurung, Shahi, Sherpa, Rokaya, Thakuri, Kshetri, Bhramin, Sarki, etc. They are Indo-Aryan and Tibeto-Burmans, speaking Nepali, and some Tibetan dialects as Kham. Most of them practice the Hindu and Buddhist religion, and have traits reflecting a mix of Tibeto-Burman and Indo-Aryan cultures. Agricultural land use focuses on cultivation of wild rice (*Chino*, *Kaguno*), buckwheat, potato, etc. on less fertile land.

The cultural and social control of high-altitude medicinal plants was studied in communities living inside the Shey-Phoksundo National Park [30]. The local economy relies on agriculture, grazing and seasonal trade. Due to low production, most of the people rely on the collection of wild medicinal plants for subsistence. They are engaged particularly in collecting medicinal herbs and raw food items as part of their traditional ventures [28]. However the collection in the Himalaya region is being increasingly driven by commercial demands from wider market [24].

## Methods

Ethnomedicinal data for wild plants traditionally used by rural inhabitants of the study area were recorded during field visits, and vouchers of the encountered plant species were collected. A total of five field visits were conducted: one in each district and two in Dolpa district (July 2001 and May 2003 for Dolpa district, September-October 2001 for Humla district, November-December 2004 for Jumla district, July-August 2003 for Mustang district). Surveys, personal interviews and group discussions as Participatory Rural Appraisal (PRA) technique [31] were applied to reveal the specific information about traditional healing practices and ethnomedicinal uses of plants. Altogether 480 (160 in Dolpa district, 110 in Humla district, 115 in Jumla district, and 95 in Mustang district) local healers, experienced aged persons and Amchi were consulted for information on folk uses of plants, which was further authenticated by cross-checking with key informants. The key informants were Amchi and experienced older persons.

Plant specimens were collected following Cunningham [32] and identified in the field up to species level whenever possible. The nomenclature follows [33-39]. All species were collected, and compared to vouchers at Tribhuvan University Central Herbarium (TUCH), and deposited in TUCH with duplicates in the King Mahendra Trust for Nature Conservation/Annapurna Conservation Area Project, Nepal. The species presented here include the most important of the total ethnomedicinal plant species, with all samples found in at least for two districts included.

## Results and discussion

Among the 529 useful plant species recorded from Dolpa district so far, more than 400 species are medicinal [40]. Of the total medicinal plant species, the most important 100 species were recorded by Lama et al. [41] and 58 species were encountered from Dunai, Juphal, Majhphal, Sahartara and Suu villages of Dolpa district [42]. The present study states that there are 107, 59, 44 and 166 species of ethnomedicinal importance in surveyed areas of Dolpa, Humla, Jumla and Mustang district respectively. Of these, 84 common species in terms of distribution and folk use were selected to enumerate their ethnomedicinal properties. The 84 species belong to 75 genera and 39 families. *Allium wallichii, Cordyceps sinensis, Dactylorhiza hatagirea*, and *Rheum australe *were especially common in study area. A total of 21 species were most common in three districts and 59 in two districts. The genera *Aconitum, Allium, Arisaema, Berberis, Corydalis, Gentiana, Hippophae, Juniperus *and *Rhododendron *each possessed two species with ethnomedicinal use. Labiatae was the most medicinally important family with five species used, followed by Araceae, Compositae, Liliaceae, Polygonaceae, Ranunculaceae, Scrophulariaceae and Umbelliferae, each contributing four species.

The plant parts used for medicinal preparations were bark, flower, fruit, leaf, root, rhizome, tuber, seed, shoot, resin, wood, etc. (Table 2). In some cases the whole plant was utilized. The most frequently utilized plant part was the root/rhizome/tuber (26.15%), followed by leaf (23.84%) and flower and fruit (21.53%). The high importance of the underground part was attributed to having high concentrations of bioactive compounds [43]. The largest number of remedies (21.70%) were used to treat respiratory tract infections (asthma, cold, cough, fever, headache, pneumonia, sinusitis, etc.) while 19.92% remedies were taken to cure gastro-intestinal problems (cholera, gastritis, intestinal pain, stomachache, etc.), 7.82% remedies were used for skeleto-muscular problems (arthritis, fracture, rheumatism, sprain, swelling, etc.) and dermatological problems (scabies, skin disease, etc.), 4.27% for ENT (ear, nose and throat) problems, and to a lesser extent for cuts and wounds, dental, cardiovascular system, circulatory system, etc. Plants were also important as tonic, astringent, anthelminthic, insecticide, incense stick, appetite stimulant, antidote, etc. The preparation methods included decoction, juice, oil, paste, powder, extract, smoke, and raw (unprocessed). The majority of remedies were prepared as juice (29.52%), followed by raw (19.04%), paste (16.19%), extract (13.33%), decoction (11.42%), powder (6.66%), etc.

**Table 2:** Illness, plant parts in use and mode of preparations

**SN**	**Illness**	**Plant parts in use**	**Mode of preparations**
1	Fever (26)	Root/rhizome/tuber (34)	Juice (31)
2	Cough and cold (15)	Leaf (31)	Raw (20)
3	Indigestion (12)	Flower and fruit (28)	Paste (17)
4	Diarrhea (11)	Whole plant (10)	Extract (14)
5	Tonic (10)	Seed (8)	Decoction (12)
6	Skin disease (10)	Shoot (7)	Powder (7)
7	Cuts and wounds (10)	Bark (7)	Oil (3)
8	Appetite stimulant (9)	Wood (3)	Smoke (1)
9	Headache (9)	Resin (2)	
10	Dysentery (9)		

Figure in parenthesis represents the number of species in use

Since the populations of the study areas belong to different ethnic groups, there are disparities and commonalities in the way of employing the same plant species and preparing remedies. *Jurinea dolomiaea *is used as incense and ethnomedicine for diarrhea and stomachache in Dolpa district, whereas it is employed only as incense in Humla district. Seed of *Juniperus indica *is eaten in Dolpa to get relief from kidney problems, whereas its leaf juice is taken for cough, cold and paralysis in Humla district. Fruit juice and seed coat of *Juglans regia *are employed to treat to wounds in Jumla district while the paste from bark is applied for arthritis and hair growth in Dolpa district. A complete list of all plants with scientific and vernacular names, usage, collection number and location is given in the additional file 1.

The observations from the present survey need to be substantiated with pharmaco-chemical studies in order to evaluate the effectiveness of the herbs and preparations used. However, for some species, there is evidence in the literature that the mode of use by the local people is likely to be effective. Application of rhubarb root paste in diarrhea and dysentery coincides to the pharmaco-chemical properties because of the purgative and astringent effects [44].

Medicinal and aromatic plants play a vital role in the life support systems of contemporary civilization by serving the purpose of maintaining good health and well being of mankind. The plants in the Himalayas grow very slowly and cannot live elsewhere, and have substantial importance to the hill and mountain peasants. Despite gradual socio-cultural transformation in the Himalaya regions [45], local communities still possess invaluable knowledge of plants and their uses. Large numbers of people are engaged in collection of medicinal and aromatic plants. It was found that herders are the main collectors of high altitude medicinal plants, which they harvest from alpine meadows and pastures [46]. Though local people do not have much scientific knowledge on sustainable harvesting of medicinal plants, they are familiar with habitat specificity and biology of plants [47]. Ethnoecological knowledge and practices of a given area are important to consider in sustainable management of Himalayan medicinal plant resources [48].

The plants are widely used as resource for grazing in the Himalayas. Rotational grazing of livestock and selective harvesting mainly applied by Amchis were only the sustainable management approaches aimed at constraining pressures. Though the practices were noticeable in all districts, former was important Dolpa, Jumla and Humla districts and the later was most in Dolpa and Mustang districts.

## Conclusion

In the Himalaya, most of the people follow Buddhism and Bon religion and they have strong belief and faith on traditional herbal medicinal practice for health treatment and therefore, the conservation of medicinal plants is not only vital to their livelihood but also has immense cultural significance to them [49]. Medicinal plants are now found growing sporadically in forests/pastures as well as in village groves. Uses of the plants and their produce/products from nearby forests by rural people are common because there is no alternative method to adopt. Forests have commercially exploitable medicinal species, which if manage properly can serve a sustainable income sources for local communities. An urgent need, therefore for conservation of medicinal plant species and their habitats and indigenous knowledge, is required. Ethnoecological knowledge, plant life forms and growth patterns are imperative to consider for management of Himalayan medicinal herbs [50].

## Declaration of competing interests

The author(s) declare that they have no competing interests.

## Authors' contributions

All authors share the contributions to this MS. The fieldwork for data collection was carried out by Ripu M Kunwar, Bal K Nepal, Hari B Kshhetri and Sanjeev K Rai. Data analysis and manuscript preparation were conducted by all authors.

## Supplementary Material

Additional file 1 List of plants, vernacular names, indigenous uses and distribution Click here for file
