# Supplementary material for: Ethnomedicine in Himalaya: a case study from Dolpa, Humla, Jumla and Mustang districts of Nepal
Source: J Ethnobiol Ethnomed. 2006 Jun 2;2:27. doi: 10.1186/1746-4269-2-27 (PMC1513199; doi:10.1186/1746-4269-2-27)
Supplement: Additional file 1 — List of plants, vernacular names, indigenous uses and distribution [file 1746-4269-2-27-s1.pdf]

Additional file 1: List of plants, vernacular names, indigenous uses and distribution.

Family	Scientific Name	Vernacular Names	Uses	Coll. #	Location
Aceraceae	*Acer acuminatum* Wall. ex D.Don	Firfire, Tilailo (Np)	Juice from wood is employed for gastritis.	D43	D, H
Amaryllidaceae	*Allium wallichii* Kuntz	Jimnak (Am), Ban lasun (Np)	Decoction of leaves is for indigestion, abdominal pain and applied as tonic.	D8	D, H, J, Mus
Araceae	*Acorus calamus* L.	Tsu dak (Am), Sweet flag (Eng), Bojho (Np), Bhadra (Sn)	Small dried rhizome is used to treat toothache, cough and cold, throat pain. It is also used as appetite stimulant.	D5	D, J
Araceae	*Arisaema flavum* (Forsk.) Schott	Tanso, Dhawa (Am), Arisaema (Eng), Tinchu (Km), Banko (Np)	Tubers are insecticidal and anthelminthic in properties. Extract from tuber is taken for scabies, stomachache and swelling. Flower juice is considered for regulating menstruation.	D423	D, Mus
Araceae	*Arisaema jacquemontii* Blume	Dhawa (Am), Dolo (Km), Banko (Np)	Juice from leaves and tubers is anthelminthic in properties. It is used in stomachache, earache and toothache.	M14	D, Mus
Araceae	*Typhonium diversifolium* Wall. ex Schoot	Dwa (Am), Ruga sag (Np)	Ingestion of tuber without proper cooking causes pain and swelling of tongue. It is used in stomach pain and cold. Shoots are taken as vegetable.	J80	D, J
Berberidaceae	*Berberis aristata* DC.	Kyerwa (Am), Barberry (Eng), Duktser (Km), Chutro (Np), Rasanjan (Sn)	Bark and root decoction is used in jaundice, fever, skin disease, eye disease, headache, diarrhea and dysentery.	D18	D, H
Berberidaceae	*Berberis lycium* Royle	Kyerwa (Am), Chimma, Dhuk, (Km), Muse chutro (Np)	Juice from leaves, fruits and bark is used in eye diseases.	M19	D, Mus

Berberidaceae	*Podophyllum hexandrum* Royle	Ol me so (Am), Gophal kangadi, Sinmedo, Balagu (Km), Laghupatra (Np, Sn)	Fruit is used in menstrual problems and gynoelogical disorders. Root paste is taken in worm infection and control bleeding.	J62	D, J
Betulaceae	*Alnus nepalensis* D.Don	Ram syang (Am), Alder (Eng), Utis (Np)	Bark juice is used in headache, fever and chest pain.	H9	D, H
Betulaceae	*Betula utilis* D.Don	Takpa (Am), Birch (Eng), Bhojpatra (Np)	Leaf decoction is used in diuretics. Young bark and shoot juice stops bleeding. Resin is taken in bile disease.	D788	D, H, Mus
Boraginaceae	*Arnebia benthamii* (Wall. ex G.Don) I.M. Johnston	Dimok (Am), Muksi (Km), Maharangi (Np)	Root juice is taken for hair tonic, headache and blood disorders.	M15	D, J, Mus
Cannabaceae	*Cannabis sativa* L.	Soma nagpo (Am), Hemp (Eng), Bhango, Ganja (Np), Bhanga (Sn)	Oil extracted from seed is used in backbone pain, insomnia, gastritis, cold, lymph disorder and pimples.	D22	D, H, J
Caryophyllaceae	*Drymaria cordata* (L.) Willd. ex Roem. & Schult.	Lightning weed (Eng), Abijalo (Np)	Root extract is taken in gastritis.	J35	D, J
Caryophyllaceae	*Silene gonosperma* (Rupr.) Bocquet.	Dhumanema, Sugu (Am)	Leaf and flower paste is used in mental disorder.	M73	D, Mus
Compositae	*Anaphalis triplinervis* (Sims) C.B. Clarke	Taygun, Ganda bata (Am), Buki phul (Np)	Leaf, stem and flower decoction is taken in chest pain.	M10	D, Mus
Compositae	*Arctium lappa* L.	Jisung (Am), Great burdock (Eng), Kurro, Tine (Np)	Seeds are digestive and used in gall and kidney stone.	D12	D, Mus
Compositae	*Jurinea dolomiaea* Bioss.	Sila poe (Am), Kalthaple, Dhupjadi (Np)	Root juice is used in diarrhea and stomachache. Plant is used as incense.	D492	D, H
Compositae	*Taraxacum officinale* F.H. Wigg.	Bitterwort (Eng), Gobre phul, Tuknu (Np)	Plant juice is considered to use in insomnia.	J76	D, J
Cupressaceae	*Juniperus indica* Bertol.	Shug pa, Lha suk (Am), Black juniper (Eng), Dhupi (Np)	Juvenile leaf juice controls cough, cold and paralysis. Leaves and branches are burnt for incense. Fruits and	D46	D, H, Mus

			seeds are used in kidney diseases.		
Cupressaceae	*Juniperus squamata* Buch.-Ham. ex D.Don	Sebaro, Shug pa, Shuk tser, Poma tser (Am), Dhupi (Np)	Paste from young leaf shoot is used in fever and skin disease. Fruits are digestive in function.	D47	D, Mus
Elaeagnaceae	*Hippophae salicifolia* D.Don	Tarbu namtar (Am), Seabuckthorn (Eng), Dalechuk, Torbu, Tora, Chichi (Km, Np), Ashuka (Sn)	Fruit juice is used in swelling of gum, wounds, dysentery, intestinal pain, fever, cough and cold and toothache. Ripe fruit is taken as tonic and appetizer. It is also taken in tuberculosis and diabetes.	D777	D, J, Mus
Elaeagnaceae	*Hippophae tibetana* Schlecht.	Tarbu satar (Am), Seabuckthorn (Eng), Tirshuk (Km), Dalechuk (Np)	Fruits are considered as appetite stimulant.	D44	D, Mus
Ephedraceae	*Ephedra gerardiana* Wall. ex Stapf	Takwe (Am), Ephedra (Eng), Kagcharo, Tshe (Km), Simtari, Salajari, Somlata (Np), Soma (Sn)	Stem and fruit juice is used in cuts, wounds, altitude sickness, indigestion, diarrhea and bleeding. Plant smoke is taken during eye troubles. Leaf powder is taken to control asthma.	D36	D, Mus
Equisetaceae	*Equisetum diffusum* D.Don	Chu tshe (Am), Horsetail (Eng), Ukchoe (Km)	Juice from whole plant stops bleeding and relieves chest pain.	M37	D, Mus
Ericaceae	*Rhododendron anthopogon* D.Don	Balukarpo (Am), Balu (Km), Dhup Sunpati, (Np)	Flower is used as herbal tea. It is effective in gastritis, common cold, indigestion and diuretic.	J67	D, H, Mus
Ericaceae	*Rhododendron arboreum* Smith	Balumarpo (Am), Rhododendron (Eng), Laligurans (Np), Rohitaka (San), Kalma (She)	Flower is used in throat pain, diarrhea, burns and scalds. Young leaf is chewed to get relief from headache. Plant is also used as fish poison.	H68	D, H
Gentianaceae	*Gentiana nubigena* Edgew.	Pangyen ngonpo (Am)	Leaf and flower paste is applied in throat pain, chest	M38	D, Mus

			pain, pimples and lung fever.		
Gentianaceae	*Gentiana robusta* King ex Hook. f.	Kyiche karpo (Am), Tite (Km, Np)	Paste from leaves and flowers is appetite stimulant and useful in bile disorder and altitude sickness.	D39	D, Mus
Gentianaceae	*Swertia nervosa* (G.Don) C.B. Clarke	Tite (Np)	Plant decoction is used in fever and pneumonia. Young leaf juice is appetite stimulant.	D783	D, J
Geraniaceae	*Geranium wallichianum* D.Don ex Sweet	Ligador (Am), Crane's bill (Eng), Raktamul (Km)	Root extract is taken in fever, cough and cold, joint pain, swelling menstrual problem and dysentery.	H40	D, H
Hypocreaceae	*Cordyceps sinensis* (Berk) Sacc.	Yar tsa gun bu (Am), Cordyceps (Eng), Jiwanbuti, Yarsagumba (Km, Np)	Whole plant is used as tonic, expectorant and stimulant. It is also used in fever, diarrhea and rheumatism.	D26	D, H, J, Mus
Juglandaceae	*Juglans regia* L.	Tar ka (Am), Himalayan walnut (Eng), Okhar (Np), Ashotaka (Sn)	Bark paste promotes hair growth and is taken to cure arthritis, skin diseases and toothache. Seed coat is taken for healing wounds.	D45	D, J
Labiatae	*Ajuga lupulina* Maxim.	Zintig (Am), Buggle weed (Eng), Khangsu (Km)	Paste from leaves, flowers and fruits is taken for skin disease, fever, sinusitis and epilepsy.	J6	D, J
Labiatae	*Dracocephalum heterophylum* Benth.	Jibkar, Tiyangku (Am), Atunmetok (Km)	Plant juice is used in liver disorders, fever and toothache.	M34	D, Mus
Labiatae	*Origanum vulgare* L.	Mag yokpa (Am), Marjoram (Eng), Kair (Km), Ram tulsi (Np)	Leaf and flower juice is applied in cold and cough, toothache and indigestion.	J55	D, J
Labiatae	*Thymus linearis* Benth.	Mak tokpa, Jhisyang, Gnarsing (Am), Ghodamarcha (Km, Np)	Leaf juice is used in toothache. It is appetite stimulant, and taken as herbal tea.	D497	D, H, Mus
Labiatae	*Lamiophlomis rotata*	Ta pak (Am, Km)	Plant powder heals damaged	M50	D, Mus

	(Benth. ex Hook.f.) Kudo		bones, joint pain and sprain.		
Leguminosae	*Caragana gerardiana* Royle	Tanglang, Ji tser, Zomoshing (Am), Thaling (Km)	Wood is substitute of *Santalum album*.	D23	D, Mus
Leguminosae	*Trigonella emodi* Benth.	Busuhang, Kote (Am)	Juice of leaf and flower is taken in cough, cuts and wound. Excess consumption induces worm in stomach.	M79	D, Mus
Liliaceae	*Allium hypsistum* Stearn	Koje, Jim nak, Gokpa (Am), Jimmu (Np)	Leaf juice is used in cough and cold and taken as appetite stimulant.	J7	H, J, Mus
Liliaceae	*Asparagus filicinus* Buch.-Ham. ex D.Don	Nyeshing (Am), Rapuk (Km), Ban kurilo, Satawari (Np)	Tubers are used as astringent. Tuber paste is taken in diarrhea, dysentery, fever, cough and cold, lactation and skin disease. Root powder is used as tonic. Fruits are taken for pimples.	D16	D, J, Mus
Liliaceae	*Paris polyphylla* Smith	Satuwa (Np)	Decoction of root is anthelminthic and antiseptic in properties. Root extract induces wound healing and it acts as antidote of Aconite poisoning.	D498	D, H, J
Liliaceae	*Polygonatum cirrhifolium* (Wall.) Royle	Ranye (Am), Solomon's seal (Eng), Kheraulo (Np), Mahamedo (Sn)	Root juice is used as tonic and is taken in fractures.	D63	D, Mus
Morchellaceae	*Morchella conica* (L.) Pers.	Morel (Eng), Mathyaura (Km), Guchi chyau (Np)	Plant is taken as vegetable. It is also considered as tonic and used in fever.	D52	D, H, J
Orchidaceae	*Dactylorhiza hatagirea* (D.Don) Soo	Wang lak (Am), Lovha (Km), Hathejadi, Panchaule (Np), Airalu (Sn)	Juice from rhizome is taken in cuts, wounds and gastritis.	D31	D, H, J, Mus
Orchidaceae	*Malaxis muscifera* (Lindl) Kuntze	Wongril (Am), Rakpo (Km)	Rhizome is tonic in nature.	M51	D, Mus

Papaveraceae	*Corydalis cashmeriana* Royle	Tongri zilpa (Am), Ye khi (Km)	Leaf and flower juice is applied in fever and jaundice.	M28	D, Mus
Papaveraceae	*Corydalis megacalyx* Ludlow	Tongzil, Tongri serpo (Am), Rekon, Sikya (Km)	Plant paste is used in fever, liver trouble, jaundice, ulcer and blood purification.	M29	D, Mus
Parnassiaceae	*Parnassia nubicola* Wall.	Jeje lakpa (Am), Mamira, Nirmasi, Bismaro (Np)	Leaf juice is applied to treat eye problems, wounds and inflammation.	D746	D, H
Pinaceae	*Cedrus deodara* (Roxb. ex D.Don) G.Don	Himalayan cedar (Eng), Dyar, Debdar (Np), Suradaru (San)	Wood oil is used in skin disease and scabies. It is also used as antilice and antileech. The bark decoction is for fever, diarrhea and dysentery. Leaf extract is messaged to get relief from body pain.	D24	D, J
Pinaceae	*Pinus wallichiana* A.B. Jackson	Thesing, Doma (Am), Blue pine (Eng), Gobre salla (Np)	Resin is employed to treat stomachache and body pain. It is also used to cure snakebite.	D60	D, H
Polygonaceae	*Aconogonum molle* (D.Don) Hara	Nyalowa (Am), Smart weed (Eng), Chawanle, Thotne (Km, Np)	Extract of leaves and stem is taken for intestinal pain.	D4	D, H
Polygonaceae	*Oxyria digyna* (L.) Hill	Chuma tsi (Am), Mountain sorrel (Eng), Kyurmu (Km), Rakte bujo (Np)	Leaf and flower juice is useful in dysentery.	M56	D, Mus
Polygonaceae	*Rheum australe* D.Don	Chu tsa (Am), Himalayan rhubarb (Eng), Tarbu atar, Late chuk, Akchya, Mire chuk (Km), Padamchal (Np)	Root paste is applied in sprain and fractures. It is taken in chest pain, cough and cold, diarrhea, dysentery and swelling. Leaves and petiole are anthelminthic and appetite stimulant.	D702	D, H, J, Mus

Polygonaceae	*Rumex nepalensis* Spreng.	Lung sho (Am), Sheep sorrel (Eng), Shoma (Km), Halhale (Np)	Root paste cures joint injury and fractures. Seeds are useful in mouth disorders. Shoot extract is taken in constipation and sores.	D71	D, H, Mus
Primulaceae	*Androsace strigillosa* Franch.	Gyatik nakpo (Am), Metok (Km)	Leaves and flower juice is applied in fever and body swelling.	M11	D, Mus
Primulaceae	*Primula sikkimensis* Hook. f.	Sangdil karpo (Am), Syaule (Km)	Flower juice is used in fever, blood disorders and diarrhea.	M64	D, Mus
Ranunculaceae	*Aconitum bisma* (Buch.-Ham) Rapaics	Bong nak (Am), Aconite (Eng), Dug (Km), Bikhma (Np)	Dried root for nausea and vomiting, fever, stomach disorder, antidote to poisoning,	H2	H, J
Ranunculaceae	*Aconitum ferox* Wall. ex Seringe	Aconite (Eng), Bikh (Np), Vatsanaba (Sn)	Root extract is used in leprosy, fever and cholera.	H3	D, H
Ranunculaceae	*Clematis tibetana* Kuntze	Imong nak po (Am), Jhujukul (Km)	Paste from leaves, stem and flowers is used in wound, cough, cold and joint pain.	M25	D, Mus
Ranunculaceae	*Delphinium himalayai* Munz.	Atik, Atimyue (Am, Km), Atis (Np)	Root is useful in astringent. Root juice is taken in snakebite, cough, fever, liver problems and headache.	D468	D, H, J
Rosaceae	*Cotoneaster microphyllus* Wall. ex Lindley	Tsar leb (Am), Tsharin (Km), Mase, Pate (Np)	Fruit controls menstruation and bleeding.	D30	D, Mus
Rosaceae	*Princepia utilis* Royle	Dhatelo (Np), Yormang (She)	Seed oil is used in rheumatism, dysentery, fever and muscular pain.	D65	D, H
Rosaceae	*Rosa macrophylla* Lindley	Segoe fo (Am), Amdoga, Kesar (Km), Gulaf (Np)	Stem and fruit paste is taken in fever and diarrhea.	D69	D, Mus
Rutaceae	*Zanthoxylum armatum* DC.	Nepal pepper (Eng), Timur (Np), Tejswani (Sn)	Seeds is powdered and taken for liver problems. Fruits are appetite stimulant. Fruits and barks are taken in indigestion and toothache.	D84	D, J
Saxifragaceae	*Astilbe rivularis* Buch.-Ham. ex D.Don	Tholo okhti (Np)	Decoction of root is taken in dysentery.	H17	D, H, J

Saxifragaceae	*Bergenia ciliata* (Haw.) Sternb.	Gatik mukpo, Gadur (Am), Rock foil (Eng), Simtadi, Silpari, Dhungephul (Km), Pakhanved (Np), Asamaved (Sn)	Root decoction is taken in diarrhea, dysentery, fever and respiratory problems. It maintains the irregular menstrual cycle. It is also used as antiemetic and anthelminthic.	D20	D, H, J
Scrophulariaceae	*Lagotis kunawurensis* (Royle ex Benth.) Rupr.	Ba sha ka (Am), Tikta (Km, Np)	Leaf and root juice is taken in high blood pressure, blood vomiting, haematopsis and haematuria.	M49	D, Mus
Scrophulariaceae	*Neopicrorhiza scrophulariiflora* (Pennel) Hong	Hong len (Am), Katuko, Tikta (Km), Kutki (Np), Katuka (Sn)	Root paste is used in fever, gastritis, intestinal pain, cough and cold, headache, eye problem and bile disorders.	D502	D, H
Scrophulariaceae	*Pedicularis longiflora* Rudolph	Langna serpo, Lugru (Am, Km)	Whole plant extract dries up lymph fluid.	M59	D, Mus
Scrophulariaceae	*Verbascum thapsus* L.	Yuk tsin (Am), Mullein (Eng), Gunupuchh (Km), Gan puchhre (Np)	Root extract is useful in constipation and urine problem. Leaf, stem and flower juice is taken in blood disorder, cuts and wounds.	J83	D, J, Mus
Solanaceae	*Datura stramonium* L.	Mdak (Am), Thorn apple (Eng), Dhaturo (Np), Dhustura (Sn)	Extract of leaves, flowers and fruits is used in fever, pain and rheumatism.	D32	D, J
Solanaceae	*Solanum nigrum* L.	Black nightshade (Eng), Kalo bhindo (Np)	Powder of root is taken in intestinal pain.	J74	J, Mus
Taxaceae	*Taxus wallichiana* Zucc.	Sanga sung (Am), Himalayan yew (Eng), Kandeloto (Km), Loth salla (Np)	Leaf extract is applied in skin diseases and cancer. It is also used in asthma, bronchitis, joint problem and muscular pain.	D77	D, H
Umbelliferae	*Cortia depressa* (D. Don) C. Norman	Tunak yungwa (Am), Njara (Km),	Root paste is taken in kidney problem, constipation,	M27	D, Mus

		Nigale sag (Np)	throat pain and gout.		
Umbelliferae	*Heracleum candicans* Wall. ex DC.	Tukar (Am), Sukar, Chhetaro (Km)	Root powder is useful for earache. Root extract is used in joint pain, bleeding and leprosy.	M42	D, Mus
Umbelliferae	*Selinum tenuifolium* Wall.	Chawa, Chella, Tunak (Am), Ragwort (Eng), Bhutkesh, Bhatauri (Np), Kanthaparna (Sn), Rhuji (She)	Root decoction is used in diarrhea, cuts, wounds, stomachache and eye problem.	D709	D, H, Mus
Umbelliferae	*Pleurospermum dentatum* (DC.) C.B. Clarke	Gannaino (Np)	Root powder is taken with warm water in indigestion. It is taken orally for bile disorders and gall stone.	J61	H, J
Urticaceae	*Girardinia diversifolia* (Link) Friis	Chapo (Am), Himalayan nettle (Eng), Alle sisno (Km, Np)	Root paste is applied on swelling.	J41	D, H, J
Urticaceae	*Urtica dioica* L.	Stinging nettle (Eng), Polo (Km), Sisnoo (Np)	Root juice is taken in skin disease and kidney problems. It is also used as diuretics, anthelminthic and vegetable.	D81	D, H, Mus
Valerianaceae	*Nardostachys grandiflora* DC.	Drak poe (Am), Spikenard (Eng), Bhulte (Km), Jatamasi (Np), Pangbu (She), Gandhamasi (Sn)	Rhizome decoction is applied in diuretics, indigestion, leprosy, epilepsy, fever and constipation. Leaves are chewed in headache and altitude sickness.	D710	D, H, J
Valerianaceae	*Valeriana jatamansii* Jones	Nak poe (Am), Valerian (Eng), Samayo (Km), Sugandhwal (Np), Tagara (Sn)	Rhizome paste is applied in headache, sore throat, indigestion and shock. Shoot juice is useful in eye problem.	D82	D, H, J

Zingiberaceae	*Roscoea alpina* Royle	Wangla nempo (Am), Bhadauri, Kakoli, Nakali panchaunle (Np)	Root decoction is used as tonic. It is applied in headache and fever.	J70	D, J

Few vernacular names were taken from IUCN Nepal[3,5], Lama et al[41] and Ghimire et al[49]

Am = Amchi, Eng = English, Km = Kham, Np = Nepali, She = Sherpa, Sn = Sanskrit,

D = Dolpa District, H = Humla District, J = Jumla District and Mus = Mustang District
